# Design Optimization and FE Analysis of 3D Printed Carbon PEEK Based Mono Leaf Spring

**DOI:** 10.3390/mi10050279

**Published:** 2019-04-26

**Authors:** Amir Kessentini, Gulam Mohammed Sayeed Ahmed, Jamel Madiouli

**Affiliations:** 1 Department of Mechanical Engineering, College of Engineering, King Khalid University, P.O. Box 9004, Abha-61413, Asir, Saudi Arabia; gmsa786@kku.edu.sa (G.M.S.A.); jmmadiouli@kku.edu.sa (J.M.); 2 Laboratory of Electromechanical Systems (LASEM), National Engineering School of Sfax, University of Sfax, Route de Soukra km 4, Sfax 3038, Tunisia; 3 Engineering School of Monastir, Laboratory of Thermal Research and Thermodynamics of Industrial Processes LRTTPI, University of Monastir, Monastir 5019, Tunisia

**Keywords:** design, composite materials, leaf spring, automotive, 3D printing, carbon PEEK, optimization, finite element analysis (FEA)

## Abstract

In this research work, design optimization and static analysis of a 3D printed based carbon PEEK (poly ether ether ketone, reinforced with carbon) polymer composite mono leaf spring was done using finite element analysis. Comparative study of leaf springs of a Dodge SUV car has been made by using 3D printed carbon PEEK. The main objective of this work is to optimize the design and material parameters, such as fiber diameter, fiber length, percentage volume of fibers and orientation angle of fibers in 3D printed based material with a mono polymer composite leaf spring. The effects of these parameters were studied to evaluate the deflection, bending stress, spring rate, stiffness and von Mises stress under different loading conditions. Furthermore investigation has been done to reduce the weight of leaf springs and claimed the 3D printed based leaf springs have better load carrying capacity. Thus an attempt has been made in this regard and we selected the 3D printed carbon PEEK in developing product design and material selection for minimum deflection and bending stress by means of response surface optimization methodology for an efficient leaf spring suspension system. The 3D printed carbon fiber polymer composite has three different percentage volume fractions such as 30%, 50%, and 60%. The selected carbon PEEK has 0°, 45°, and 90° fiber orientations. Finite element based analysis has been performed on 3D printed carbon PEEK material to conclude the optimized design parameters and best possible combination of factors affecting the leaf spring performance.

## 1. Introduction

**Figure 1:**
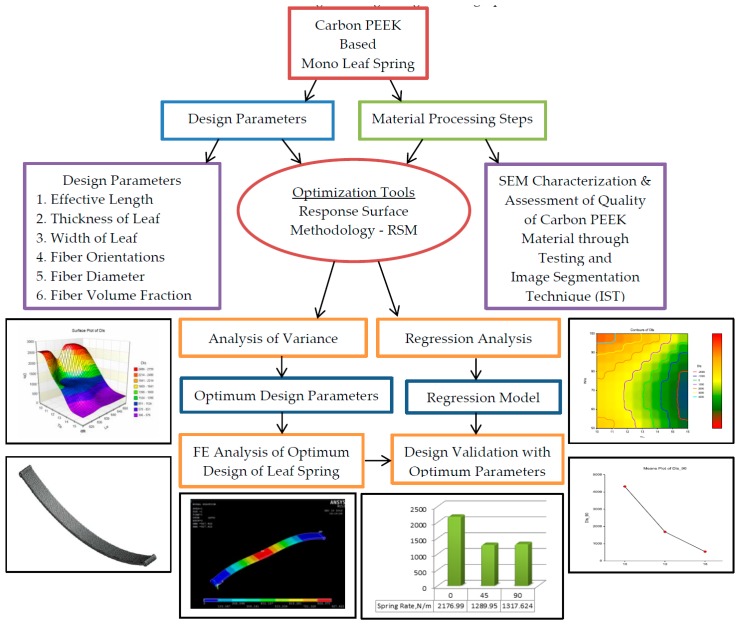
The procedural steps for design optimization.

A leaf spring is a simple form of spring which is usually called ‘balestra’, commonly used in automobile suspensions. Leaf springs are made from flat plates which are called leaves; it is one of the oldest forms of spring and serves a damping function. The friction in interleaf provides the damping action. The vehicles must have a good suspension system so that a good ride and human comfort can be achieved. There are many types of springs which are available in a vehicle suspension system such as helical spring, conical and volute spring, torsion spring and laminated leaf spring. The leaves are given an initial cambere and when the load is applied they will tend to straighten. The leaf spring has two eyes which are front and rear eye; the front eye and rear eye is found at the front and rear end of the master leaf respectively. When the vehicle comes across a jump or projection on the road surface, the wheel moves up and leads to reduced deflection of the spring and changes the length between spring eyes. The ability of the leaf spring is to absorb and store an amount of energy that ensures comfortable operation of a suspension system. In previous studies, harmonic and modal analysis for a multi-leaf spring for different existing conventional metals has been done using finite element analysis (FEA), software ANSYS 12.1 and compared with theoretical values [1]. A similar study on design of a mono composite leaf spring with varying thickness has been performed using static analysis in FEA based software ANSYS14.5 [2]. The effect of friction and hysteresis loops on the spring rate was determined experimentally and the results claimed hysteresis loop characteristics make loading asymmetric [3,4]. Several studies have been conducted on leaf spring analysis, such as deflection and stress analysis using the finite element method (FEM). The vertical stiffness and stress analysis conducted is based on the vertical loading of the leaf spring as well as the stress concentration zone was explored and equivalent stresses induced in parabolic leaf springs were determined by considering geometry and under variable loading conditions [4,5]. In one of the recent studies the strain gauge technique was used in evaluation of stresses in the master leaf with and without extra full length leaves. The durability of the leaf spring was evaluated by testing and the simulation method was based on accelerated fatigue life testing [6]. Replacement of steel leaf springs with composite materials has been tested for mono composite leaf spring for the same load carrying capacity and stiffness; the main aim of their study was to determine the composite material based leaf spring elastic strain energy and strength-to-weight ratio compared to those of steel. It has been found that there is a reduction in the weight of the leaf spring without a reduction in load carrying capacity and stiffness [7]. In another work, the clamping effect is modeled through an equivalent force at the tip and calculated through post processing of experimental results. The master leaf spring is modeled as a curved cantilever beam under a tip concentrated load and analysis is carried out to evaluate deflections under static loading conditions. The static and dynamic parameters of the suspension system were identified and optimized to obtain a better performance of leaf springs [8,9]. Experimental work was carried out on various types of leaf springs such as glass fiber reinforced plastic, carbon fiber reinforced epoxy, glass fiber reinforced epoxy, glass and carbon fibers reinforced epoxy, and graphite fiber reinforced epoxy are the most used composite materials [10,11]. A spring model has been considered for estimation of deformation and stress by using the finite element technique by considering a layered parabolic leaf spring and has been experimentally validated [12]. Artificial neural networks have been applied to evaluate the optimum stresses induced in the leaf spring under the influence of span and camber of the leaf spring [13,14,15]. Though more research work has been done on conventional leaf spring suspension, in the present work an attempt has been made to explore the possibilities and capabilities of additive manufacturing in the development of a leaf spring using 3D printed carbon PEEK materials. The present research work searched for an opportunity and fills the gap with highly efficient leaf spring materials. Structural analysis of a leaf spring has been performed using properties of new 3D printed materials. Results are presented for optimum design parameters and results are evaluated for two leaf springs using 3D printed based carbon PEEK materials. A leaf spring has been modeled as a cantilever beam with different parameters such as length, thickness, width, fiber orientation, fiber diameter, Percentage volume fraction of fiber fill, and different load conditions. The 3D printed specimens were developed an ARGO machine, as shown in Figure 1. Also optimization has been done to evaluate the best possible combination of the geometry parameters of leaf spring and material parameters of carbon poly ether ether ketone (PEEK). The damping capacity of carbon PEEK has better absorption of strain and vibration energy leads to reduced dynamic vibration noise. Three dimensional printed carbon PEEK can be produced and with desired geometry with weight reduction, higher stiffness, and load carrying capacity. Initially, in the present study, properties of carbon PEEK, such as tensile strength, modulus, density, and strength-to-weight ratio are studied. Carbon PEEK has a high elastic modulus in the direction of orientation, strength, corrosion resistant, good fatigue, and adhesion properties when compare to conventional steel. It has become an appropriate choice due to these advantages for the suspension of Dodge SUV (Sport Utility Vehicle) vehicle leaf springs. 

## 2. Research Methodology

The proposed methodology is used to evaluate the capabilities of 3D printed carbon PEEK material in suspension leaf springs described by the flowchart shown in Figure 1. In summary of the work, the design of experiment (DoE) is devised and specimens are developed by the fused filament fabrication process. The regression analysis based models are developed based on tensile tests and are validated. The main objective of the present study is to build robust empirical models to predict deflection and stresses induced under different geometry and material parameters. The DoE should allow the corresponding relations between these characteristics and main process parameters to be fitted. It is an economical DoE for three controlling factors such as fiber orientation, fiber diameter and percentage fiber volume fractions used in the 3D printing process, as shown in Table 1. This DoE requires three levels for each factor in order to investigate a large range of design parameters.

## 3. Materials and Methods

### 3.1. 3D Printed Carbon PEEK Materials for Leaf Spring

The selection of suitable materials for leaf spring design involves more design constraints and material characteristics considerations. In this research work 3D printed carbon PEEK polymer composites were considered and analyzed for optimum design using SolidWorks simulation Xpress software to determine finite element based results. The carbon PEEK has higher strength-to-weight ratio, natural frequencies, and fatigue resistance when compared to conventional leaf spring strength. The properties of the samples were taken from the supplier, i.e., Roboze, Bari, Italy [16]. Figure 3 presents the schematic representation of the 3D printing Process with fiber orientation (a) 0°, (b) 45°, and (c) 90°. In the present work 10%, 30% and 50% percentage fiber volume fraction of carbon fibers were selected. Polymers are notable for their unmatched lightweight properties as a result of their density values ranging from 0.8 to 1.6 g/cm^3^ [17]. Commercially available polymers such as those with carbon fiber reinforced polyamide and super polymers such as carbon PEEK, possess high strength to weight ratios. Polymers possess high mechanical properties, and area light aluminum alloy replacement, due to their low density. Three dimensional printing materials proved to be a vital material in automotive, aerospace and industrial applications, [18,19]. They offer high mechanical strength and thermal properties, such as carbon PEEK, and can be a better material selection for metal replacement in extreme applications [20,21,22]. Customized product development is easily possible with improved renewable design, durability, and strength in cutting edge 3D printed materials. [23,24,25,26,27]. In the present work, carbon PEEK was selected for its remarkable rigidity and thermal stability due to the addition of fused carbon fibers in the PEEK matrix with desired fiber diameter, fiber length, fiber orientation, and volume fraction of fibers. These additions were possible in 3D printed carbon PEEK with customized product development, improved stiffness, load carrying capacity, compressive strength, and capability in maintaining higher degree temperatures (HDT). The parameters used for 3D printed carbon PEEK are given in Table 1.

**Figure 2:**
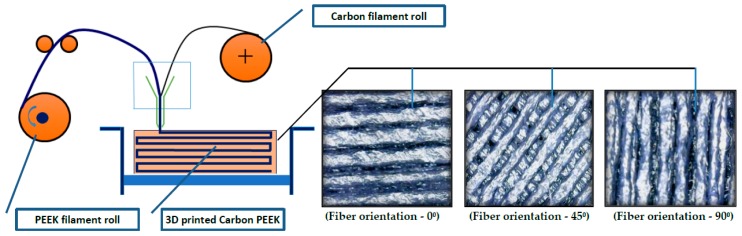
3D printing process with fiber orientations.

**Figure 3:**
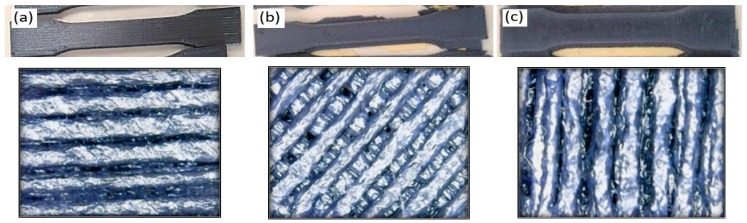
(**a**) Fiber orientation—0°; (**b**) Fiber orientation—45°; (**c**) Fiber orientation—90°.

Poly ether ether ketone (PEEK)-based polymer composite materials have controlled combinations of properties achievable with FFF (fused filament fabrication) technology of 3D printing such that combination of at least two different conventional materials is possible. PEEK matrix materials play a vital role in many sectors due to their chemical-physical properties and light weight. In the automotive sector, PEEK composites allow us to meet the need for strength and weight reduction. Polymer composites are not affected by corrosion and are used as coatings to create protective barriers [29,30,31]. Schematic representation of the FFF process is presented in Figure 2 and Figure 3 shows carbon PEEK tensile specimens with different fiber orientations. 

### 3.2. 3D Printed Carbon PEEK Materials and the FFF Method

PEEK with its remarkable capability of resistance to aggressive acids and unsurpassable chemical resistance can replace metal alloys even in severe environments, allowing for cost reduction in maintenance. Chemical compatibilities are presented in grades in Table 2. 

**Figure 4:**
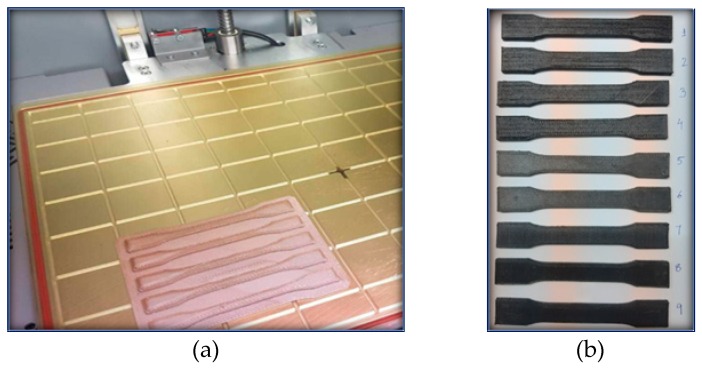
(**a**) Standard test specimen on ARGO machine bed: (**b**) 3D printed CPEEK specimens.

In the present research work, the fused filament fabrication (FFF) 3D printing technique has been used for the production of PEEK and carbon PEEK standard specimens for tensile and bending tests. The customized and conceptual prototyping to production of functional and structural components was possible with added advantages of manufacturing time and costs [33,34,35]. Carbon PEEK material was conceived and adapted to the FFF 3D printing extrusion process. The FFF process parameters are given in Table 3. The 3D printed FFF technology utilizes the Beltless system which offers accuracy, precision, and repeatability of the 3D printed products [36,37,38]. The beltless system utilizes gear teeth to ensure smooth movement, tolerance (25-microns), positioning, accuracy and precision of the high viscosity polymer extruder (HVPE) equals 0.020 mm [39]. The HVPE extruder has a confined narrow channel to accelerate high viscous polymers with desired fiber parameters, and a controlled optimal temperature to increase the printing speed of polymers [40]. A custom-made FFF ARGO-500, Roboze 3D printer was used to produce the specimens of carbon PEEK polymer composites filled with continuous or discrete carbon fibers. The extruder is the heart of FFF technology which provides power to melt the material at the desired point and spread over the entire surface of the domain [41]. In Table 3, specifications of the FFF process machine are mentioned [42,43]. The ARGO-500 3D printer, as shown in Figure 4, supplied with a chassis built by electro-coated designed to control excess vibration, and internal fittings are made from anti-corrosion Al-6082, AISI-303 stainless steel, and are chrome plated. The moveable extruder head equipped with an induction heater, that precisely locates the nozzle over the stationary heated bed can be maintained to 250 °C. The machine printing platform surfaces were electro-galvanized and heated up to 100 °C.

## 4. Design of Leaf Spring

**Figure 5:**
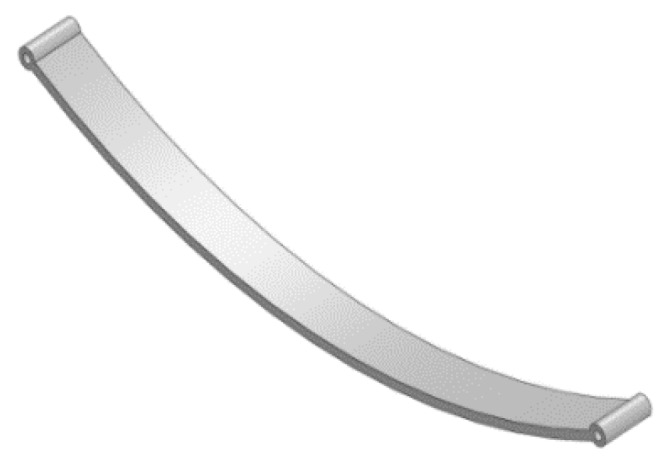
3D modeling of mono leaf spring using SolidWorks CAD software.

Three-dimensional CAD modeling of the leaf spring based on the dimension obtained from analytical calculations, was created with the help of SolidWorks CAD modeling software. The 3D model of the mono leaf spring using SolidWorks CAD software is shown in Figure 5. Finite element analysis done using ANSYS 17 deflection and stress. The Dodge SUV car selected for this study and the vehicle currently uses the leaf with center bolt, and U-clamp on the master leaf. 

## 5. Mathematical Modeling of a Mono Leaf Spring

**Figure 6:**
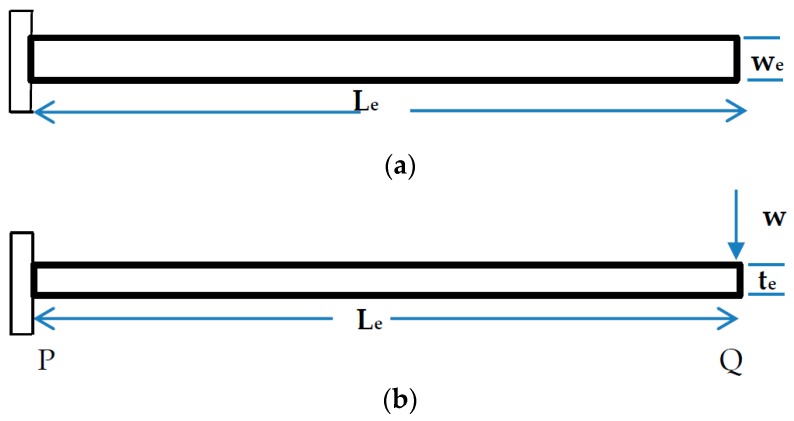
(**a**) Plan view of cantilever model of the leaf spring; (**b**) Elevation view of cantilever model of the leaf spring.

The mathematical modeling is a crucial task to understand or predict the real situation of the designed components. In this case, different loads or stresses which would be applied on the leaf spring could be considered. According to the literature, the main common mechanical stress, which affects the life of the leaf spring, is the shock produced by static loading, road irregularities, braking, and cornering during driving. In this case, the leaf spring was loaded with a static load and during analysis this type of loading was also considered. The shape of the leaf spring used for the analysis, is shown below in Figure 6a,b. The leaf spring behaves like a cantilever beam and the static analysis was done considering it as a cantilever beam. Since the leaf spring was mounted on the axle using U- bolts firmly, then the leaf spring counted as a double cantilever beam with a load W at the free end of the leaf spring and length L. The cantilever beam was highly exposed to both bending stress and transverse shear stress. Now the mathematical modeling can be derived, taken from the cantilever beam nature. This plate may be used as a flat spring. Let: t = thickness of plate, we = width of plate, and Le = length of plate or distance of the load W from the cantilever end.

The maximum bending moment at the cantilever end P is bending moment $M_{P}=W_{e}\times L_{e}$, section modulus Z=wete26, Bending stress $\sigma_{b}=\frac{6W_{e}L_{e}}{{bt}^{2}}$, and maximum deflection $\delta_{\max}=\frac{4\times W_{e}l_{e}^{3}}{{Ew}_{e}t_{e}^{3}}$

## 6. Design Optimization

### 6.1. Response Surface Methodology

The development of the deflection and stress induced in leaf spring models is performed according to response surface methodology (RSM). The prediction of a relationship between a response of interest, $Y_{d}$ and various associated control variables (design and process parameters) denoted by *x*_1_, *x*_2_… *x_q_*. The general form of the second-order model includes linear, quadratic and interaction effects, and are given by design of experiments by Montgomery [43] as follows:

(1) $$Y_{d}=b_{0}+\sum_{p=1}^{q}b_{p}x_{p}+\sum_{p=1}^{q}b_{pp}x_{p}{}^{2}+\sum_{p<r}^{r}b_{pr}x_{p}x_{r}+\epsilon$$

where *b*_0_ is the intercept, *bi*, *bij*, and *bii* are the regression coefficients, *k* denotes the number of control factors (*k* = 3 in the present study), and *ε* is a random experimental error. In order to determine the regression coefficients, experimental runs were carried out with respect to the DoE, as shown in Table 1. The chosen levels of the control factors can be represented by the following matrix;

(2) $$D=\left[\begin{array}{ccc} x_{11} & \ldots & x_{1q}\\ \vdots & \ddots & \vdots \\ x_{n1} & \ldots & x_{nq} \end{array}\right]$$

where *x_pi_* denotes the *p*-th setting level of control factor *xi*, *p* = 1, 2… *n*; *n* is the number of runs in the DoE (*n* = 9 in the present study). Let *y* = (*y*_1_, *y*_2_, *y_n_*)^*T*^ denote the response values corresponding to the *l*-th setting of *x_p_* = (*x_p_*_1_, *x_p_*_2_,…, *x_pn_*)^*T*^. Then, Equation (1) can be expressed as

(3) $$y_{p}=F^{T}\left(x_{l}\right)+\varepsilon_{p}$$

where (*x_p_*) is a vector function of elements that consists of linear, quadratic and cross-products of *x*_*l*1_, *x*_*l*2_, *x*_*ln*_. The quantity *εl* denotes the random experimental error for the *l*-th run. Considering all the experimental runs in the DoE of matrix D (Equation (2)), where *X* is a matrix of order *n* × *p*, *l*-_th_ row of which is (*x*); Equation (3) can be expressed in matrix form as

(4) y=Xγ+ε

where *ε* = (*ε*_1_, *ε*_2_, …, *ε_n_*)^*T*^; and *β* is a vector of p unknown coefficients of the model (Equation (1)). Assuming that *ε* is a random experimental error possessing a zero mean with a variance–covariance matrix given by *σ*^2^, the ordinary least-squares estimator of *γ*, denoted by $\dddot{\gamma }$ an then be determined as follows

(5) $$\dddot{\gamma }={\left(X^{T}X\right)}^{-1}X^{T}y$$

It is worth noting that the general form of the second order model (Equation (1)) includes ten regression coefficients (*p* = 9). This number depends on the significance of the control factors and their level of interactions. However the RSM based models involve linear, quadratic, and interactions effects, these effects may not be significant and must be ignored. In the present work an analysis of variance (ANOVA) was performed to determine the effects of these control factors. 

### 6.2. Regression Analysis

**Figure 7:**
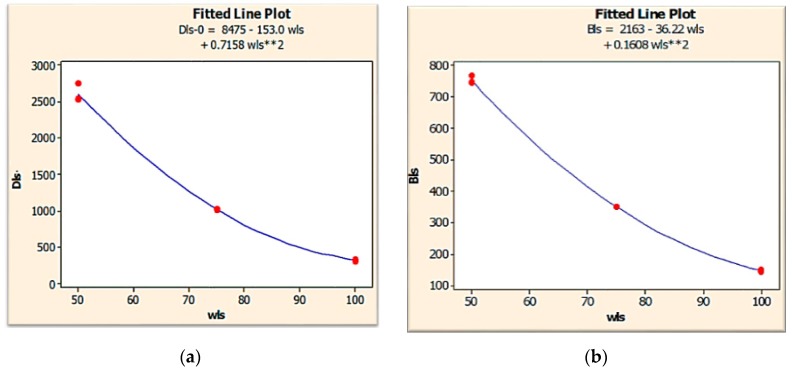
Variation of deflection and bending stress for different width.

Regression analysis was conducted based on the response surface methodology using the carbon PEEK data corresponding to the carbon PEEK material fiber diameter, fiber orientation and geometrical parameters in order to construct the predictive regression model for evaluating the effect of these parameters on deflection and von Mises stress. Regression analysis has been performed to develop the mathematical models and has been fitted to predict the main aspects of deflections in leaf springs mentioned above. The coefficients of each term in these predictive regression models are given in Table 4. The table also presents the results of the ANOVA in order to explore the significance of carbon PEEK material parameters and their interactions. Results show that all the variance analyses (P-test) at 95% confidence were found to be significant (*p*-values < 0.05) for deflection predictive models. The predictive ability of the models for this variable is significant, as the adjusted coefficients of the determination value is 95 %. Conclusively, the developed models are statistically relevant and can be applied with a high level of confidence to predict deflections in the range of the design parameters tested. *p*-values under 0.05 also show that fiber diameter and percentage fiber volume fraction were found to be significant for von Mises stress. These results suggest the possibility of properly selecting the material process conditions for designing leaf spring specimens with specific design characteristics. Figure 7, shows the variation of deflection and bending stress for different widths.

### 6.3. Multiple Regression Analysis 

The standard deviation of the regression coefficients of the equation was found to be 362.37. The estimated regression equation for different fiber orientations:

Dls = −430.5 + 447.3 × te − 100.1 × W + 5.6 × Le

The optimum values for the dimensions of the leaf spring are presented in Table 5 and the difference between FEA results and regression analysis are found to be within the permissible value of 4%. The comparison has been made for the 0° fiber orientation.

**Figure 8:**
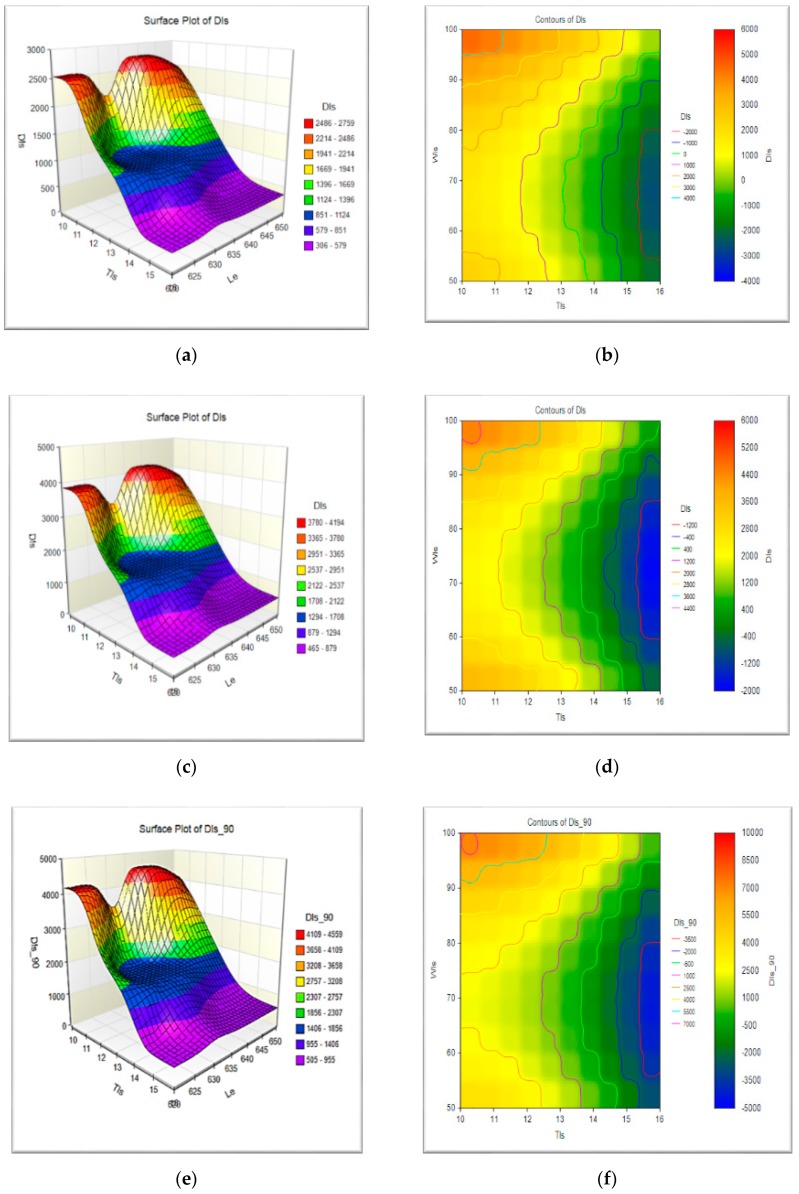
(**a**) Surface plot in fiber 0° orientation (**b**) contour plots for the deflection in fiber 0° orientation, (**c**) surface plot in fiber 45° orientation (**d**) contour plots for the deflection in fiber 45° orientation, (**e**) surface plot in fiber 90° orientation (**f**) contour plots for the deflection in fiber 90° orientation.

Surface plots and contour plots for different fiber orientations are presented in Figure 8a–e, these plots are the graphical calculators to predict the deflections for the different design parameters like thickness, width and length of leaf springs. It is a graphical calculator for different design features. The different color represents the variation of the deflections of the leaf spring. The green zone was found to have the optimum design features. 

## 7. Finite Element Analysis

**Figure 9:**
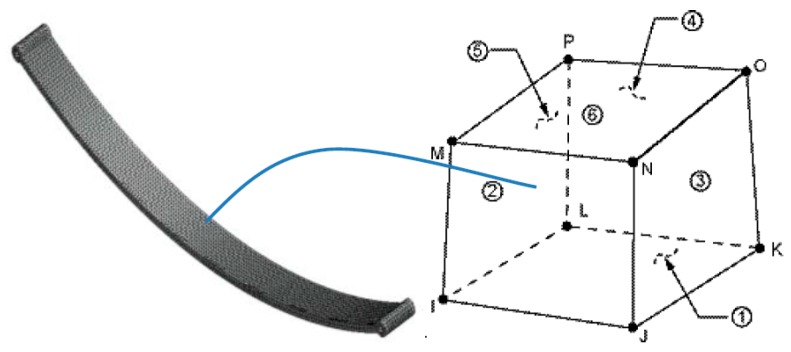
Brick eight node solid 185 meshed model of a mono leaf spring.

The geometry and node locations for the element are shown in the figure. SOLID185 is a homogeneous structural solid geometrical element shown in Figure 9. The element has eight nodes and orthotropic material properties. Separate meshing software also exists for complex geometries such as HYPERMESH but in ANSYS 15, the size of mesh can be controlled using the SIZE CONTROL option and by selecting PICKALL for the entire geometry, then each SOLID185 is used for a 3D mesh model of solid structures. It is defined by eight nodes having three degrees of freedom at each node- translations in the nodal x, y, and z directions. The element has plasticity, hyper elasticity, stress stiffening, creep, large deflection, and large strain capabilities. It also has mixed formulation capability for simulating deformations of nearly incompressible elasto-plastic materials, and fully incompressible hyper elastic materials. The SOLID185 structural solid is suitable for modeling general 3-D solid structures. 

## 8. Results and Discussions

### 8.1. Effect of Total Leaf Span

**Figure 10:**
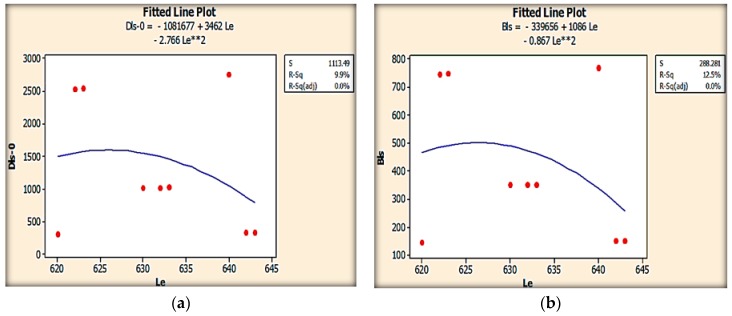
Variation of (**a**) deflection and (**b**) bending for total leaf span.

Effective length of the leaf spring is an important factor for perfect design of the leaf spring under tensile loading conditions and for a load carrying capacity with fluctuating loads. The variation of deflection and bending stress are presented in Figure 10. The carbon fibers of the leaf spring stretched under tensile loading conditions and proved to be effective with zero fiber orientation. Minimum deflections and bending stress were found to be minimum for 643 mm effective length. 

### 8.2. Effect of Percentage Volume Fraction

**Figure 11:**
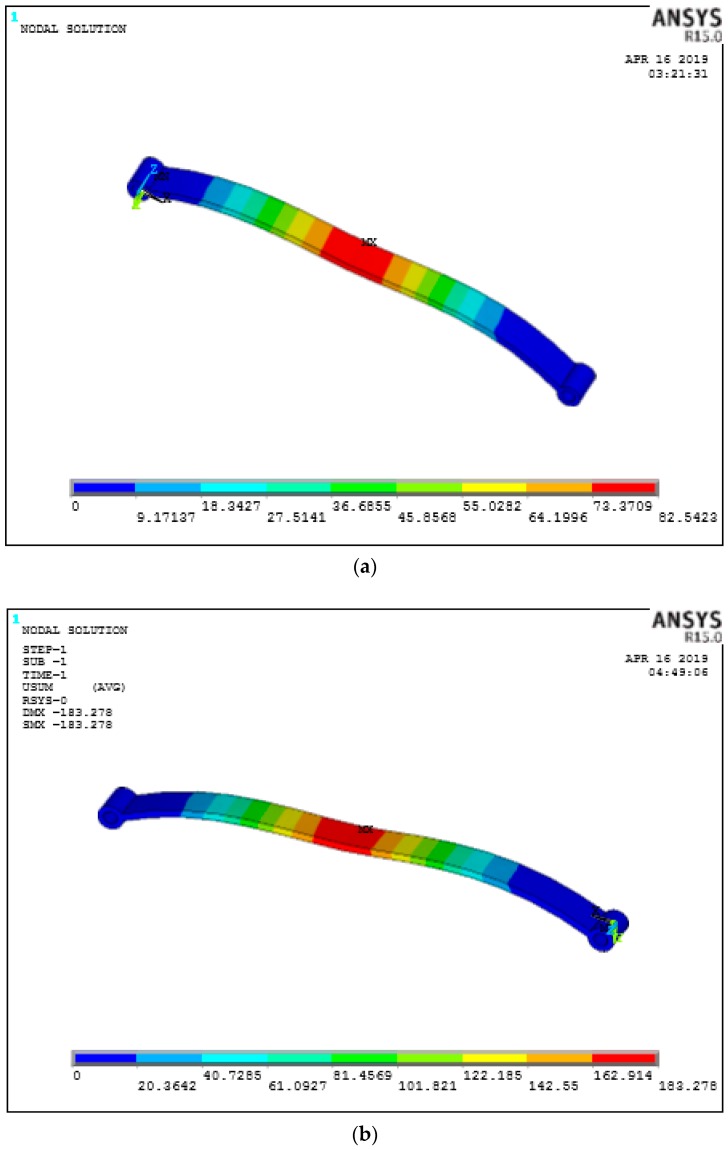

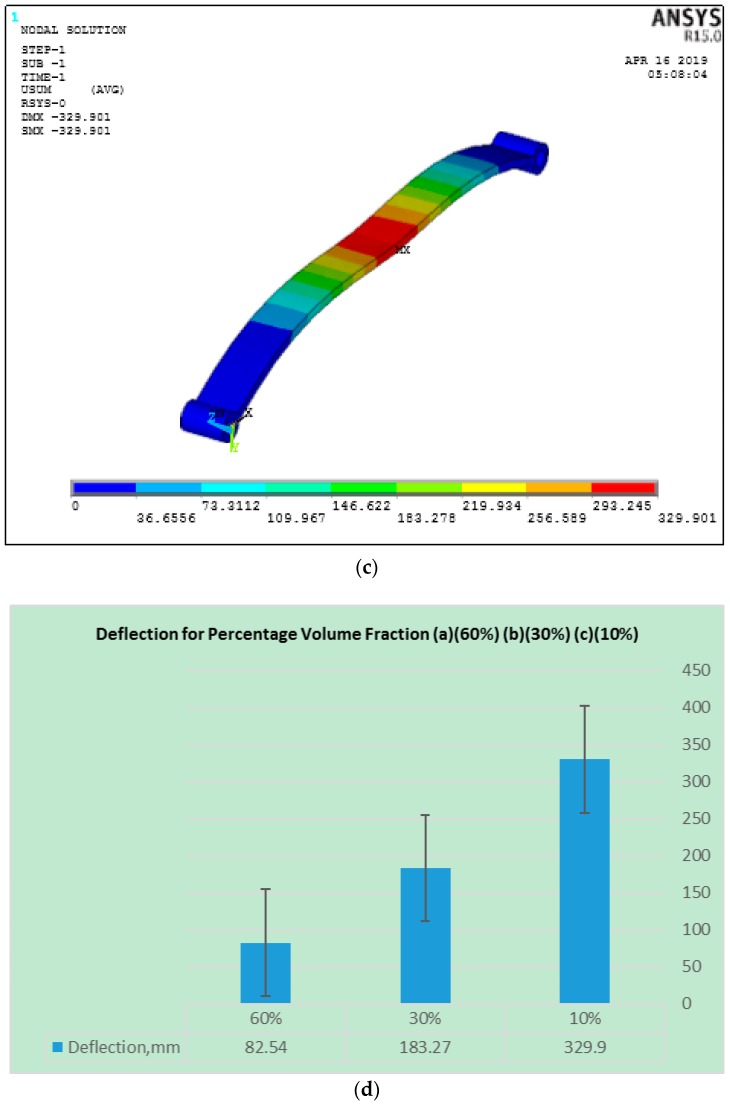
Deflection for percentage volume fraction at 0 Deg orientation only (**a**) 60% (**b**) 30% (**c**) 10% (**d**) deflections.

**Figure 12:**
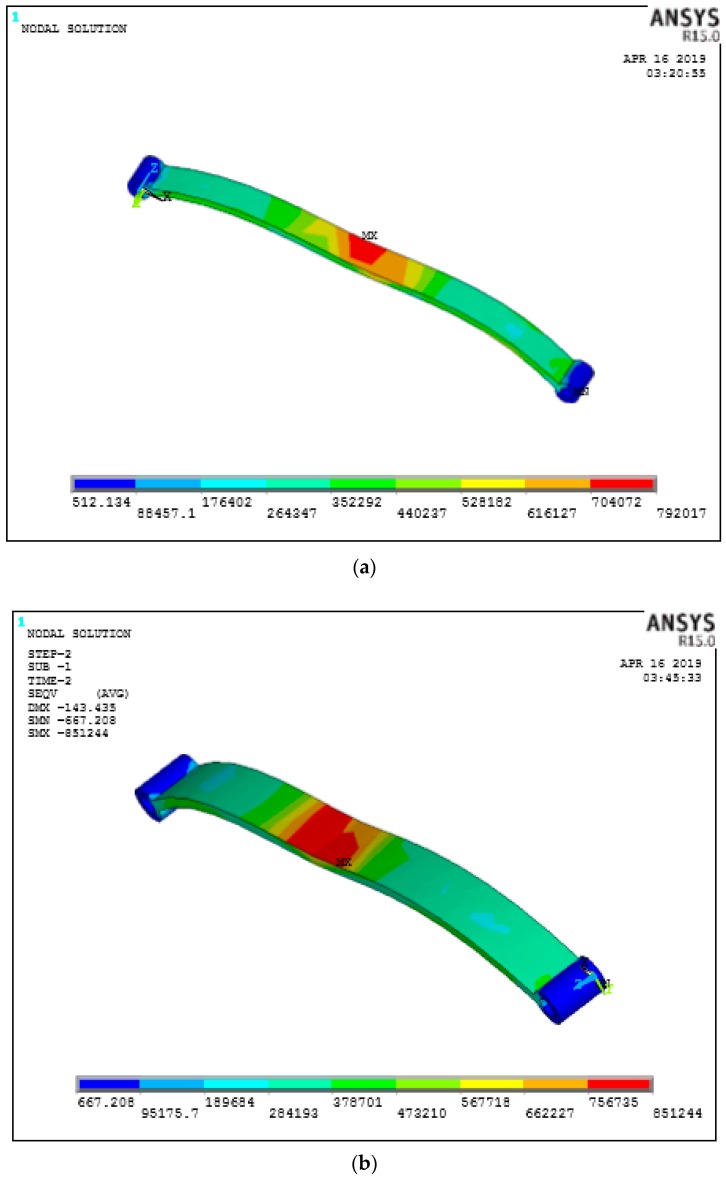

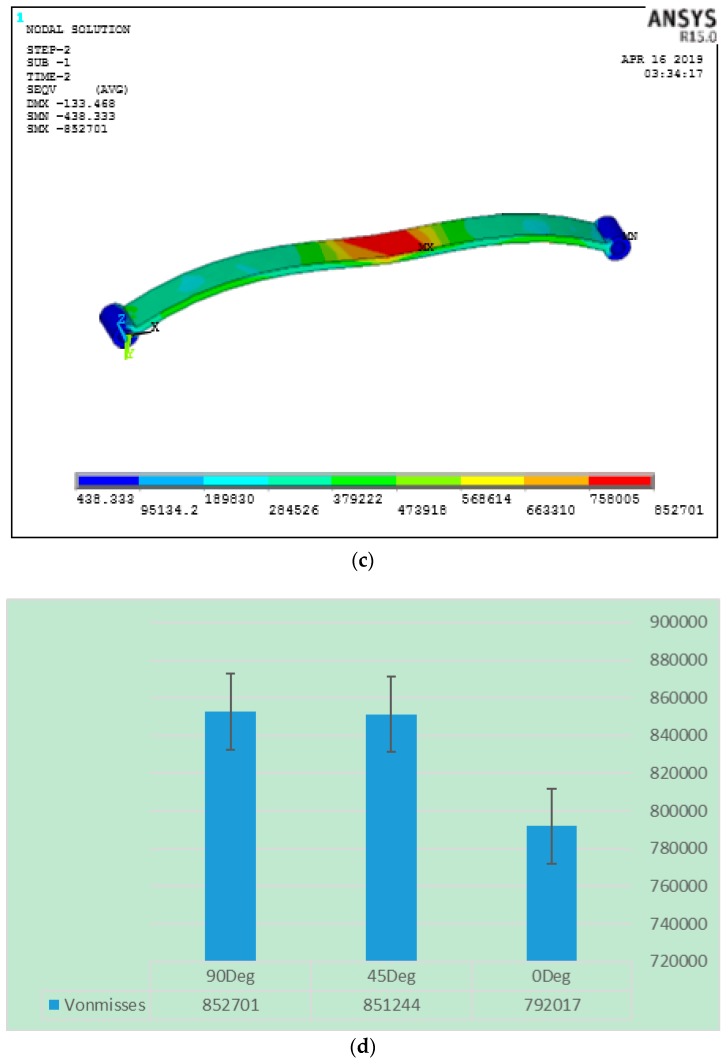
Von Mises stress for different fiber orientations (**a**) 0° (**b**) 45° (**c**) 90° (**d**) Vonmises Stresses.

Fiber volume fraction in a PEEK matrix plays a vital role in terms of strength for load carrying capacity. In the structural analysis of the leaf spring 10%, 30%, and 60% percentage fiber volume was considered and 60% with fiber 50 µm in length and 0 degrees since it has less deflection as mentioned previously and was found to have less deflection under the loading conditions as shown in Figure 11a–d. A similar trend has been found in stress as shown in Figure 12a–d it has less induced stress in the case of the 60% fiber volume fraction.

### 8.3. Effect of Fiber Orientation

**Figure 13:**
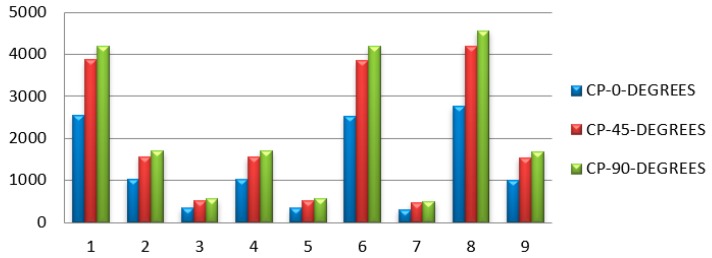
Comparison of deflection with different fiber orientation.

**Figure 14:**
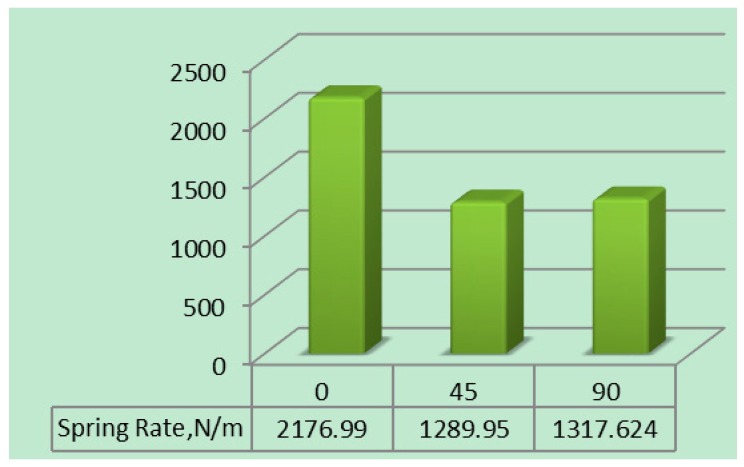
Spring rate of leaf spring for different fiber orientations.

Table 6 represents the maximum deflection and bending stress with different carbon PEEK fiber orientations. Fiber orientation with zero degrees has less deflection as shown in Figure 13. The spring rate of the carbon PEEK has larger values when compared to the other orientations that signifies the capability of the leaf spring, which is more in the case of fiber orientation at 0° as presented in Figure 14.

### 8.4. Effect of Fiber Diameter on Deflection

**Figure 15:**
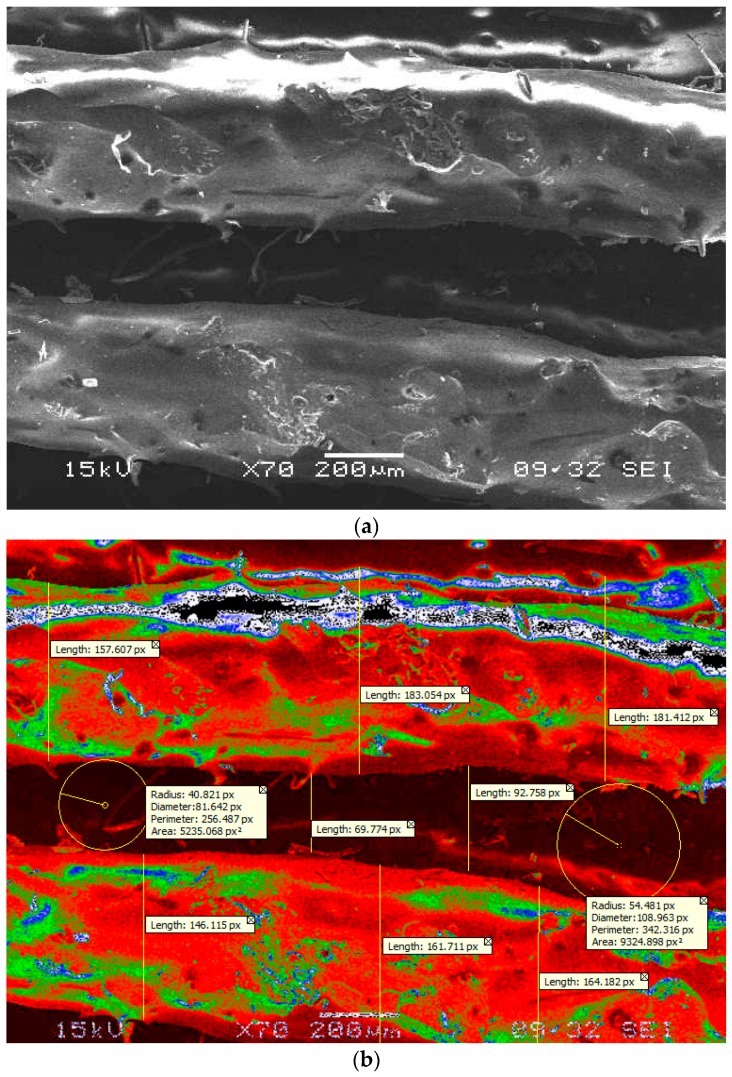
(**a**) SEM micrograph at 200 µm, (**b**) assessment of fibers at 0° by image segments analysis.

**Figure 16:**
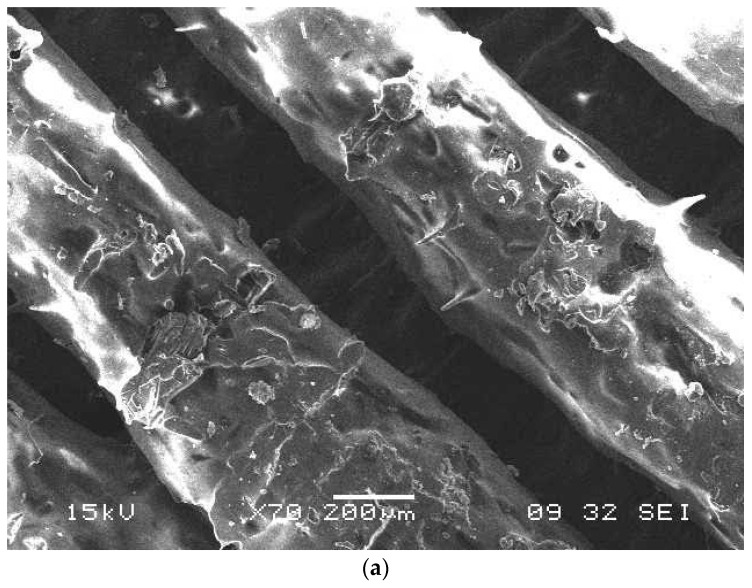

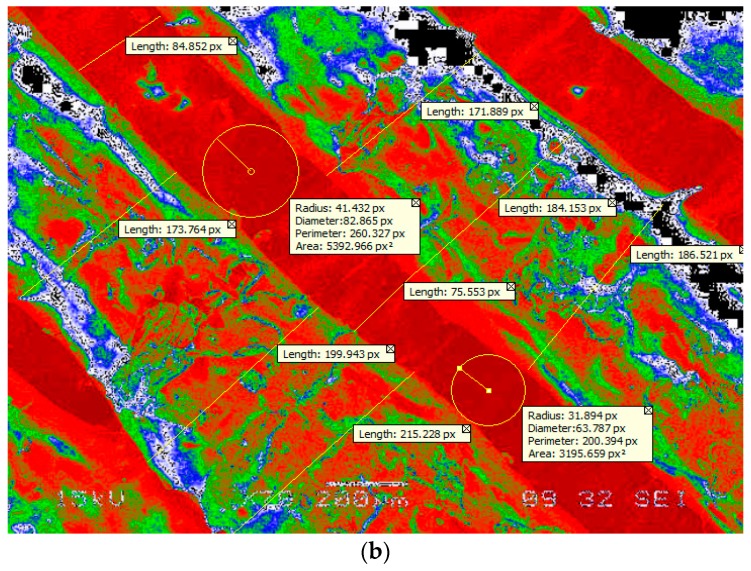
(**a**) SEM micrograph at 200 µm, (**b**) assessment of fibers at 45° by image segments analysis.

Carbon PEEK has high elastic modulus in the direction of orientation, strength, corrosion resistant, good fatigue and adhesion properties when compared to conventional steel. Carbon PEEK has become an appropriate choice due to these advantages for the suspension of the Dodge vehicle leaf spring. In the present work, 10%, 30%, and 50% percentage fiber volume fraction of carbon fibers were selected with different orientation. Firstly, the images were captured using a 500× magnification stereo microscope specially used for material characterization as shown in Figure 15a. The scaling of the pictures for determining the resolution of the pictures taken were appropriately calibrated and processed further for image analysis presented in Figure 15b and the related digimizer statistics are presented in the Table 7. The pictures captured for each carbon fiber PEEK material were distinguished based on the resolution of the images and properly assessed for image calibration. The image analysis technique was advocated in captured images for determining the fiber orientation and the clear spacing of the fiber arrangement. The image analysis technique is a process consisting of thresholding and binarization. All the captured images presented in Figure 16a for fiber orientation 450 were processed separately to determine the surface parametrics, which include the spacing and area of the fibres in the matrix as shown in Figure 16b. The image analysis studies conducted on the three types of carbon PEEK materials revealed that the fiber orientation in the direction of the preferential processing direction can provide enhanced mechanical performance. In the case of parallel orientation, i.e., zero degrees, composite fiber-matrix characteristics provide maximum tensile loading accompanied by bending resistance in orthogonal directions. The bending stress criterion suggests that maximum yielding of a ductile material imitates when the second deviatoric stress invariant reaches a critical value. This provides adequate evidence on the plastic deformation of ductile materials and very well applies for mechanical distortion/deformation in the case of leaf spring components for specific applications in a vertical energy absorption capacity. The segmented image is represented at 300 dpi with measurements in terms of pixels as 1 px = 0.084 mm. The different colors are shown in Figure 15a,b such as red, green and black on the surface of the fibers after cooling the samples. The red color is the solid fiber without any voids and the green color is the shiny smoothening surface of the fiber. The black color is the major voids that occur at the surface of the fiber during the process of laser sintering in the FFF process.

However, when the fiber alignment was at 45 degrees orientation the stress resultants were complex as the deviatroic stress components were normal to the maximum principal plane. This reveals the fact that the resulting stress during tensile pullout may sustain maximum energy absorption without yielding due to failure of the plane occurring other than the maximum principal stress direction. However, the comparative difference of varying fiber orientations provides adequate evidence in terms of the composite performance in terms of being used as an effective shock absorbing mechanism.

**Figure 17:**
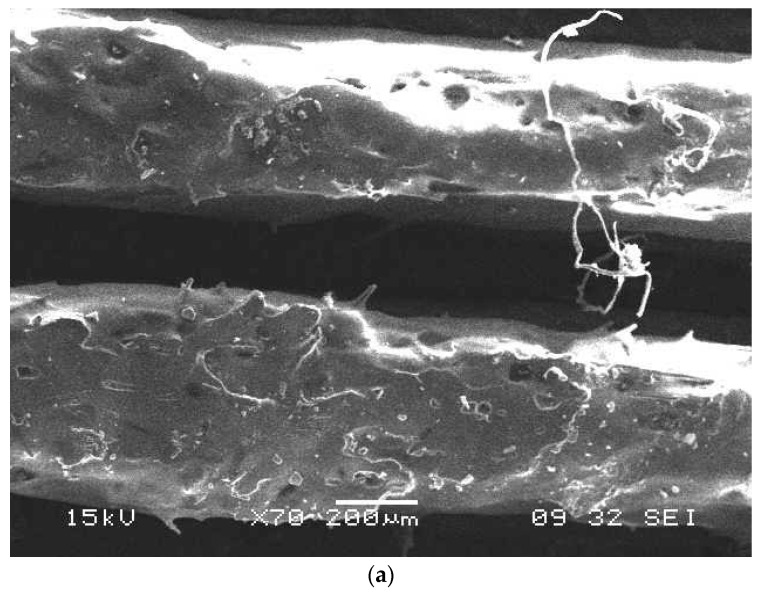

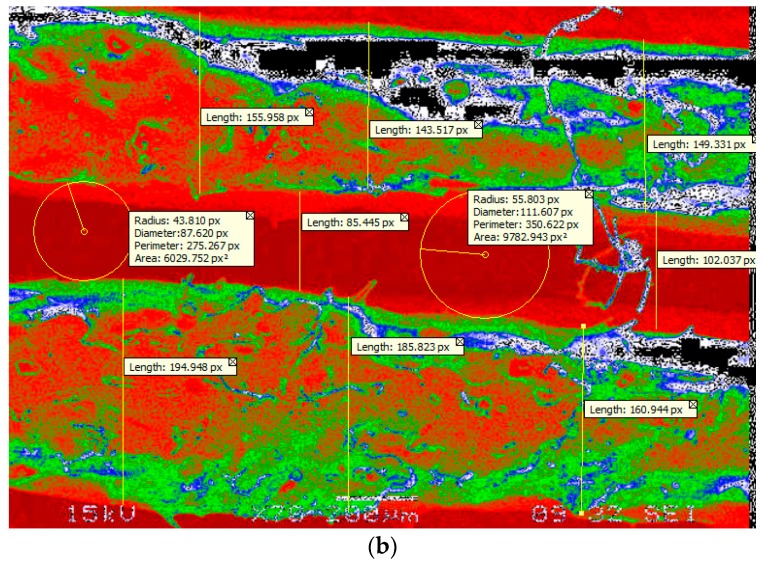
(**a**) SEM micrograph at 200 µm, (**b**) Assessment of fibers at 90^0^ by image segments analysis.

**Figure 18:**
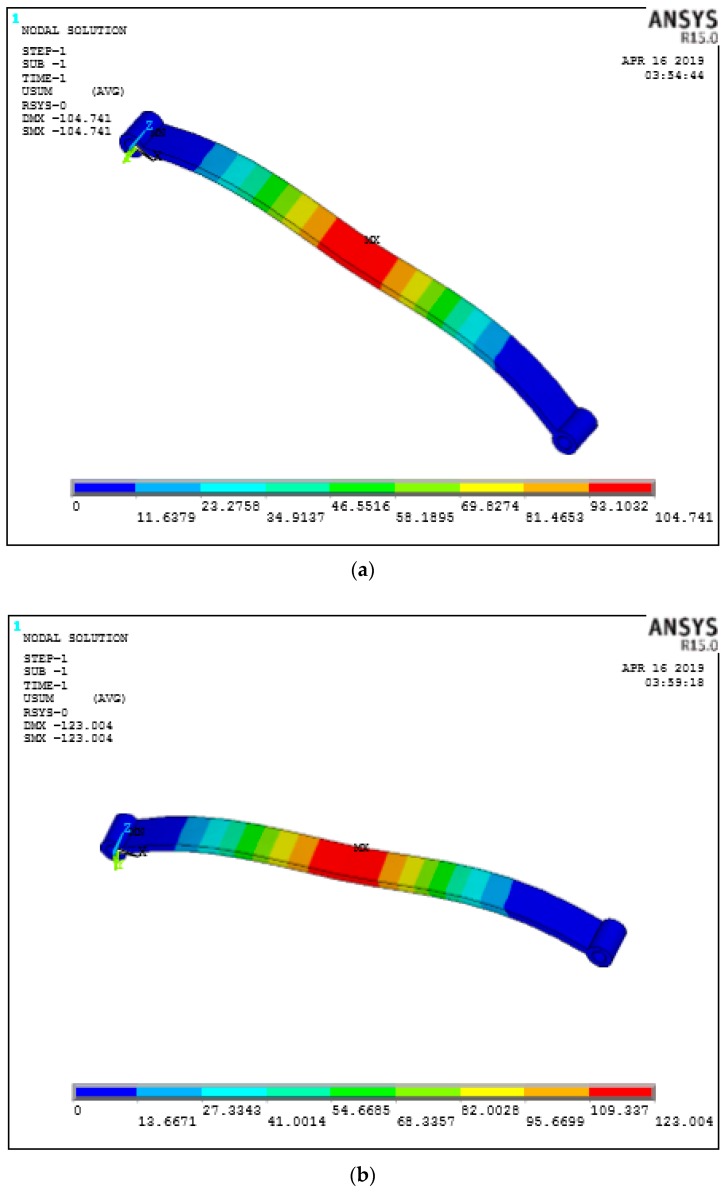

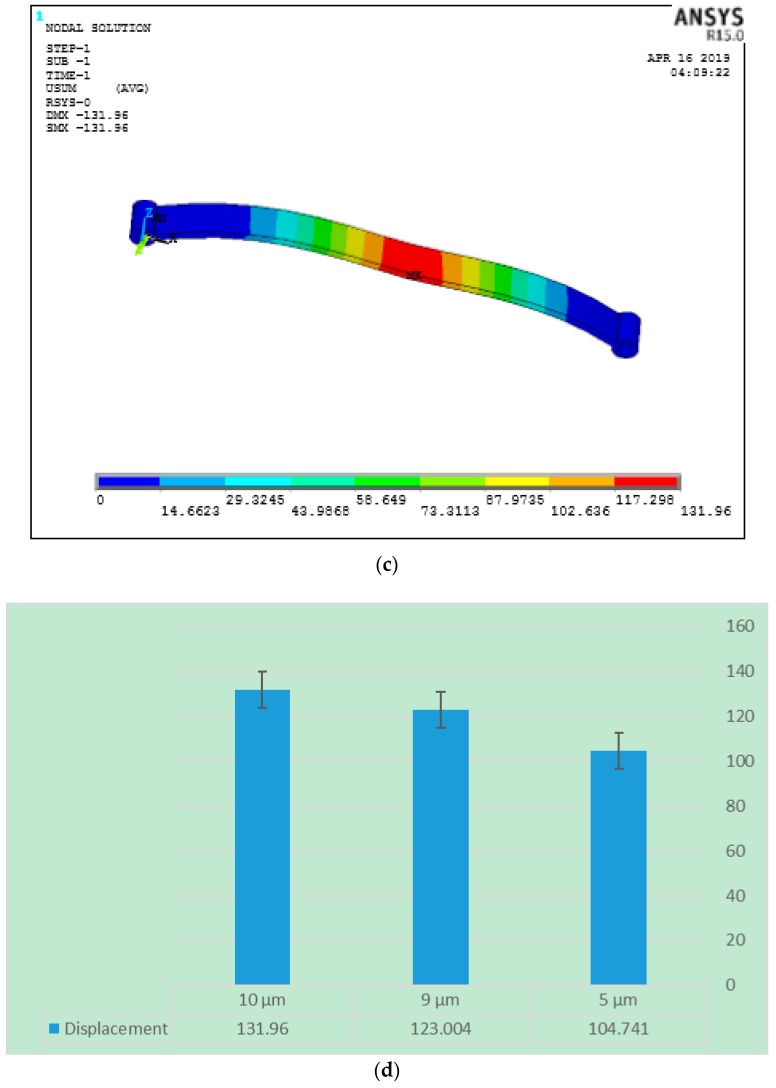
Deflections at different fiber diameters (**a**) 10 μm, (**b**) 9 μm, (**c**) 5 μm, (**d**) comparison of deflections with different fiber diameters.

When the fiber alignment was at a 90 degree orientation, the maximum stress resultants were subjected to complexity as the deviatroic stress components were perpendicular to the applied load and the possibility of maximum damage when compared to 0° and 45° fiber orientations. SEM micrograph are presented in Figure 17a and digimizer statistics presented in the Table 8 for the fiber orientation 90°, which shows the bifurcation of fibers from the pattern followed during the FFF process and causes stress raisers during loading conditions. In Figure 17b, assessment of the fiber quality is shown and the different gap between the fibers within the matrix reveals misalignment of the fibers in the matrix and can cause breakage during pullout of fibers. The finite element results show that the fiber orientation 0° proved to be beneficial in reducing deflections and the resulting stress during tensile pullout and absorbing maximum energy absorption without yielding as the maximum principal stress occurs along the fiber directions. The finite element results for deflections for different fiber diameters are presented in Figure 18a with deflection at fiber orientation 0° (b) 45° and (c) 90°. The carbon fiber with diameter 5 µm has more deflection when compare to 9 µm and 10 µm as shown in Figure 18c. The properly controlled and uniformity during the fiber processing in the FFF fiber diameter plays a vital role in reducing the deflections so that fiber pullout will be minimum. The uniform fiber diameters can be achieved with controlled temperature of the extruder nozzle during the FFF process and it also helps in proper alignment of the fibers with high accuracy.

### 8.5. Estimation of Weight Reduction

The mass of 3D printed carbon PEEK composite material leaf spring is 2.86 kg and the mass of conventional steel based leaf spring 6.5 kg. Therefore, the mass ratio is 0.44 and the percentage reduction of mass of leaf spring becomes 56.4% which means the weight of the CPEEK leaf spring is reduced by about 56.4% by replacing with conventional steel leaf spring. Comparative analysis in weight reduction has been presented in Figure and the newly designed CPEEK mono leaf spring is of lighter weight than that of the conventional steel leaf spring of the Dodge SUV car. Due to this major advantage, the CPEEK composite leaf spring helps to make vehicle suspension lightweight. Based on the results achieved the scope of future studies may include dynamic mechanical analysis and design of CPEEK mono leaf spring under dynamic loading conditions.

### 8.6. Effect of Thickness on Deflection

**Figure 19:**
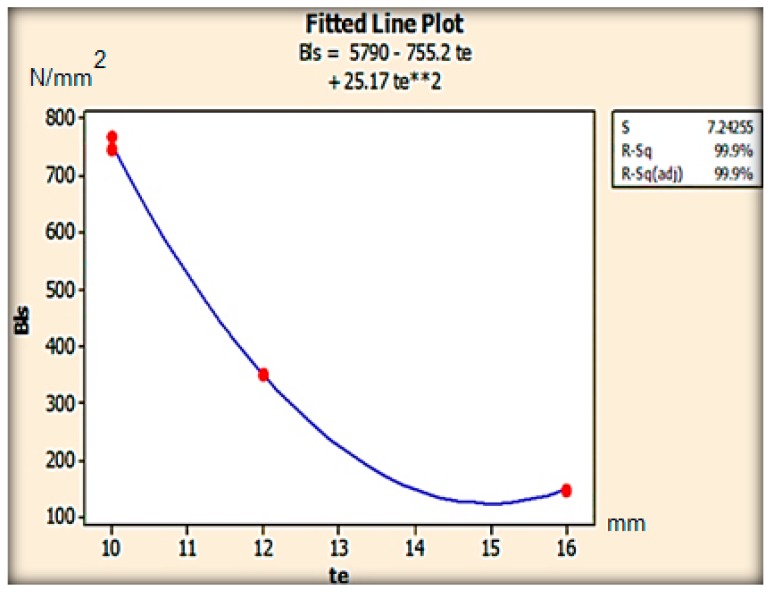
Variation of bending stress N/mm^2^, for different thickness of leaf spring.

The deflections in the leaf spring were gradually reduced as the thickness and the numbers of fiber layers were increased additively during the FFF process. The variation in bending stress was observed as shown in Figure 19 and also decreased with the thickness. The PEEK matrix provides the adhesive strength to the carbon fibers along the zero degree fiber orientation such that tensile strength of the fibers reduces the deflection during the loading conditions. The deflections were gradually decreasing with the increasing of the thickness and width. A similar trend has been observed with the bending stress with different design parameters. On analyzing the deflection and bending stress results of the carbon PEEK-based leaf spring at different thicknesses and widths it has been found that the optimum thickness value was 16 mm and width was 100 mm. Here the carbon PEEK leaf spring has a higher spring rate for the 0° fiber orientation when compare to fiber orientations of 45° and 90°. Therefore, 16 mm thick carbon PEEK-based leaf spring has more load carrying capacity with a width of 100 mm. 

## 9. Conclusions

In the present research work, a study has been made with an existing Dodge SUV leaf spring and CPEEK mono composite leaf springs under static loading conditions using FEA-based ANSYS software simulation results and analytical calculations. Finite element analysis is a powerful computational tool for simulating and analyzing complicated structural shaped bodies. An attempt has been made in this research work to utilize the 3D printing FFF technology to achieve the results based on CPEEK mono leaf spring. The present study helps in reducing physical tests and reducing the investment, time, and costs. To conclude this research work deflections, von Mises stress and suspension weight reductions were evaluated in CPEEK leaf springs against steel leaf springs for a Dodge SUV car suspension system. This was done to achieve the following. Therefore, from the optimum design and static analysis of the study, steel leaf springs replaced with a laminated carbon/epoxy composite mono leaf spring is the best one. The properties of CPEEK materials, like a high strength-to-weight ratio and more specific stiffness, are key features for the construction of an efficient, lightweight, and environmentally friendly vehicle component. The strain energy absorption capability of the CPEEK materials offers a unique combination of resistance to failure of the components and reduced weight. By using the finite element analysis with ANSYS 16, the results in this study show that a CPEEK mono leaf spring has better performance than a conventional steel leaf spring. The original methodology proposed here was used to manufacture leaf springs with controlled FFF process parameters originating from a variety of machining conditions. Various combinations of design and carbon PEEK parameters were analyzed, obtained optimum results were obtainedfor the leaf spring which has less deflection. Response surface methodology (RSM) and analysis of variance (ANOVA) enabled the development of analytical regression models capable of predicting deflections and bending stress. These were validated with finite element results obtained with various combinations of the design parameters. Regarding the validation of predictive models, the obtained results for the finite element and regression analysis results showed a good agreement with FEA results. Future works will present the dynamic analysis and its behavior of the leaf spring under dynamic loading conditions. 

## Figures and Tables

**Table 1:** 3D printed carbon PEEK material parameters [28].

	Parameters	Young’s Modulus
Fiber Diameter	5 µm	6.0 GPa
Fiber Diameter	9 µm	5.1 GPa
Fiber Diameter	10 µm	5.0 GPa
Fiber Orientation	0°	7600 MPa
Fiber Orientation	45°	5000 MPa
Fiber Orientation	90°	4600 MPa
Percentage Volume Fraction	10%	7600 MPa
Percentage Volume Fraction	30%	28,000 MPa
Percentage Volume Fraction	60%	50,000 MPa

**Table 2:** Chemical compatibility of carbon PEEK [32]. P = high, Q = medium, R = poor, S = not recommended.

Chemical Agent		Al-Alloys	AISI-316	Carbon PEEK
1	Hydrogen Peroxide	R	Q	P
2	Hydrogen Sulfide	R	R	P
3	Nitrous Oxide	Q	Q	P
4	Ammonia	Q	Q	P
5	Petroleum	S	Q	P
6	Freon	S	R	P
7	Sea Water	Q	R	P
8	Sulfurous Gas	R	R	P

**Table 3:** Specifications of the fused filament fabrication (FFF) process machine.

**Position Accuracy**	0.025 mm
**Printing Volume, X × Y × Z**	280 × 220 × 200 mm^3^
**Bed Temperature**	180 °C
**Layer Height**	0.10 mm to 0.30 mm
**Tolerance**	20–25 Microns
**Nozzle Diameter**	0.35 mm
**Nozzle Temperature**	250 °C
**Nozzle Movement Speed**	100–200 mm/min
**Nozzle Movement Pattern**	Rectilinear
**Extruder Width**	0.35 mm–0.50 mm

**Table 4:** Regression coefficients confidence intervals.

Parameters	Regression	Standard	Lower 95%	Upper 95%
Independent	Coefficient	Error	Conf. Limit	Conf. Limit
Intercept	−430.5	1489.7	−4260.1	3398.9
te	447.3	39.4	346.0	548.6
W	−100.1	4.8	−112.5	−87.6
Le	5.6	2.3	−0.4	11.8

**Table 5:** Comparison of deflections for optimum dimensions from regression and FEA results.

Thickness te, mm	Width we, mm	Length Le, mm	Regression Model, mm	FEA mm	Difference	Standard Deviation	Error of the Mean
16	100	620	243.62	257.37	5%	9.72	6.87

**Table 6:** Maximum deflection of different carbon PEEK fiber orientations.

S.No	Le	we	te	Dls-0, mm	Dls-45, mm	Dls-90, mm	Bls, N/mm^2^
1.	623	50	10	2545.31	3868.87	4205.29	747.60
2.	633	75	12	1030.04	1565.66	1701.80	351.66
3.	643	100	16	341.60	519.23	564.38	150.70
4.	632	75	12	1025.16	1558.25	1693.75	351.11
5.	642	100	16	340.01	516.82	561.76	150.46
6.	622	50	10	2533.07	3850.27	4185.08	746.40
7.	620	100	16	306.24	465.48	505.96	145.31
8.	640	50	10	2759.41	4194.30	4559.03	768.00
9.	630	75	12	1015.46	1543.50	1677.72	350.00

**Table 7:** Digimizer statistics of fiber 0° and 45° orientations in image segmentation analysis.

**Digimizer Statistics of Fiber 0° Orientation in Image Segmentation Analysis.**						
**Tool**	**Measure**	**n**	**Mean**	**SD**	**Min**	**Max**
Length	Length	8	144.57	41.34	69.77	183.05
Circle	Area	2	7279.98	2891.94	5235.06	9324.89
	Perimeter	2	299.40	60.68	256.48	342.31
	Red-Avg. Intensity	2	0.25	0.06	0.20	0.30
	Green -Avg. Intensity	2	0.00	0.00	0.00	0.00
	Blue- Avg. Intensity	2	0.00	0.00	0.00	0.00
	Radius	2	47.65	9.65	40.82	54.48
**Digimizer Statistics of Fiber 45° Orientation in Image Segmentation Analysis.**						
**Tool**	**Measure**	**n**	**Mean**	**SD**	**Min**	**Max**
Length	Length	8	144.57	41.34	69.77	183.05
Circle	Area	2	7279.98	2891.94	5235.06	9324.89
	Perimeter	2	299.40	60.68	256.48	342.31
	Red-Avg. Intensity	2	0.25	0.06	0.20	0.30
	Green -Avg. Intensity	2	0.00	0.00	0.00	0.00
	Blue- Avg. Intensity	2	0.00	0.00	0.00	0.00
	Radius	2	47.65	9.65	40.82	54.48

**Table 8:** Digimizer statistics of fiber 90° orientation in image segmentation analysis.

Tool	Measure	n	Mean	SD	Min	Max
Length	Length	8	147.25	37.62	85.44	194.94
Circle	Area	2	7906.34	2653.90	6029.75	9782.94
	Perimeter	2	312.94	53.28	275.26	350.62
	Red-Avg. Intensity	2	0.66	0.02	0.64	0.68
	Green -Avg. Intensity	2	0.00	0.00	0.00	0.01
	Blue- Avg. Intensity	2	0.00	0.00	0.00	0.00
	Radius	2	49.80	8.48	43.18	55.80

## Nomenclature

Le=Effective Length,mm.
Bls = Bending stress induced in Leaf spring
$\mathrm{Dls}-0^{{}^{\circ}}=\mathrm{Deflection}\;\mathrm{at}\;0^{{}^{\circ}}\;\mathrm{Fiber}\;\mathrm{Orientation},\mathrm{mm}$.
$\mathrm{Dls}-45^{{}^{\circ}}=\mathrm{Deflection}\;\mathrm{at}\;45^{{}^{\circ}}\;\mathrm{Fiber}\;\mathrm{Orientation},\mathrm{mm}$.
$\mathrm{Dls}-90^{{}^{\circ}}=\mathrm{Deflection}\;\mathrm{at}\;90^{{}^{\circ}}\;\mathrm{Fiber}\;\mathrm{Orientation},\mathrm{mm}$.
te=Thickness of Leaf spring , mm.
we=Width of Leaf spring , mm.
Wls=Load on mono Leaf spring, N.
$E_{f0}=\mathrm{Elasticity}\;\mathrm{Modulus}\;\mathrm{for}\;\mathrm{Fiber}\;\mathrm{orientation}\;0^{{}^{\circ}}$.
$E_{f45}=\mathrm{Elasticity}\;\mathrm{Modulus}\;\mathrm{for}\;\mathrm{Fiber}\;\mathrm{orientation}\;45^{{}^{\circ}}$.
$E_{f90}=\mathrm{Elasticity}\;\mathrm{Modulus}\;\mathrm{for}\;\mathrm{Fiber}\;\mathrm{orientation}\;90^{{}^{\circ}}$.
$E_{p}^{1}=\mathrm{Elasticity}\;\mathrm{Modulus}\;\mathrm{for}\;10\%\;\mathrm{Fiber}\;\mathrm{Volume}\;\mathrm{fraction}$.
$E_{p}^{2}=\mathrm{Elasticity}\;\mathrm{Modulus}\;\mathrm{for}\;30\%\;\mathrm{Fiber}\;\mathrm{Volume}\;\mathrm{fraction}$.
$E_{p}^{3}=\mathrm{Elasticity}\;\mathrm{Modulus}\;\mathrm{for}\;60\%\;\mathrm{Fiber}\;\mathrm{Volume}\;\mathrm{fraction}$.
$E_{f}^{1}=\mathrm{Elasticity}\;\mathrm{Modulus}\;\mathrm{for}\;\mathrm{Fiber}\;\mathrm{diameter}\;\mathrm{as}\;5\;\mu m$.
$E_{f}^{2}=\mathrm{Elasticity}\;\mathrm{Modulus}\;\mathrm{for}\;\mathrm{Fiber}\;\mathrm{diameter}\;\mathrm{as}\;9\;\mu m$.
$E_{f}^{3}=\mathrm{Elasticity}\;\mathrm{Modulus}\;\mathrm{for}\;\mathrm{Fiber}\;\mathrm{diameter}\;\mathrm{as}\;12\;\mu m$.
*υ*_peek_ = Poisson’s ratio of PEEK
*υ*_cpeek_ = Poisson’s ratio of carbon PEEK
*W*_cpeek_ = Weight of carbon PEEK based leaf spring, kg
